# Ammonia-Oxidizing Bacteria Maintain Abundance but Lower *amoA*-Gene Expression during Cold Temperature Nitrification Failure in a Full-Scale Municipal Wastewater Treatment Plant

**DOI:** 10.1128/spectrum.02571-22

**Published:** 2023-02-14

**Authors:** Juliet Johnston, Zhe Du, Sebastian Behrens

**Affiliations:** a University of Minnesota, Department of Civil, Environmental, and Geo-Engineering, Minneapolis, Minnesota, USA; b Lawrence Livermore National Laboratory, Physical and Life Sciences Directorate, Livermore, California, USA; c Center for Environmental Health Risk Assessment and Research, Chinese Research Academy of Environmental Sciences, Beijing, China; d University of Minnesota, BioTechnology Institute, St. Paul, Minnesota, USA; University of Guelph

**Keywords:** AOB, activated sludge, ammonia oxidation, nitrification, wastewater treatment

## Abstract

In this study, we explore the relationship between community structure and transcriptional activity of ammonia-oxidizing bacteria during cold temperature nitrification failure in three parallel full-scale sequencing batch reactors (SBRs) treating municipal wastewater. In the three reactors, ammonia concentrations increased with declines in wastewater temperature below 15°C. We quantified and sequenced 16S rRNA and ammonia monooxygenase (*amoA*) gene fragments in DNA and RNA extracts from activated sludge samples collected from the SBRs during the warmer seasons (summer and fall) and when water temperatures were below 15°C (winter and spring). Taxonomic community composition of *amoA* genes and transcripts did not vary much between the warmer and colder seasons. However, we observed significant differences in *amoA* transcript copy numbers between fall (highest) and spring (lowest). Ammonia-oxidizing bacteria of the genus Nitrosomonas sp. could maintain their population abundance despite lowering their *amoA* gene expression during winter and spring. In spite of relatively low population abundance, an *amoA* amplicon sequence variant (ASV) cluster identified as most similar to the *amoA* gene of Nitrosospira briensis showed the highest *amoA* transcript-to-gene ratio throughout all four seasons, indicating that some nitrifiers remain active at wastewater temperatures below 15°C. Our results show that 16S rRNA and *amoA* gene copy numbers are limited predictors of cell activity. To optimize function and performance of mixed community bioprocesses, we need to collect high-resolution quantitative transcriptomic and potentially proteomic data to resolve the response of individual species to changes in environmental parameters in engineered systems.

**IMPORTANCE** The diverse microbial community of activated sludge used in biological treatment systems exhibits dynamic seasonal shifts in community composition and activity. Many wastewater treatment plants in temperate/continental climates experience seasonal cold temperature nitrification failure. “Seasonal nitrification failure” is the discharge of elevated concentrations of ammonia (greater than 4 mg/liter) with treated wastewater during the winter (influent wastewater temperatures below 13°C). This study aims at expanding our understanding of how ammonia-oxidizing bacteria in activated sludge change in activity and growth across seasons. We quantified the ammonia monooxygenase (*amoA*) gene and transcript copy numbers using real-time PCR and sequenced the *amoA* amplicons to reveal community structure and activity changes of nitrifying microbial populations during seasonal nitrification failure in three full-scale sequencing batch reactors (SRBs) treating municipal wastewater. Relevant findings presented in this study contribute to explain seasonal nitrification performance variability in SRBs.

## INTRODUCTION

Microbial nitrification is the core component of nitrogen transformation in wastewater treatment plants (WWTPs). Coupled with denitrification (or anaerobic ammonia oxidation, anammox), nitrification removes nitrogen from wastewater to control eutrophication in the receiving water body. Nitrification includes two steps, starting with ammonia oxidation to nitrite catalyzed by ammonia-oxidizing bacteria (AOB), including in some WWTPs the recently identified comammox (complete ammonia oxidizing) bacteria of the genus Nitrospira, and ammonia-oxidizing archaea (AOA) (1). The second step is nitrite oxidation to nitrate by nitrite-oxidizing bacteria (NOB) (2). AOB catalyze ammonia oxidation using the enzyme ammonia monooxygenase (AMO). Ammonia oxidation by AMO is the first and rate-limiting step of nitrification and crucial for the stable operation of WWTPs (3).

In most WWTPs, AOB and AOA coexist, although questions remain about what conditions determine their specific abundance and activity. In many municipal WWTPs, AOB are more abundant than AOA by several orders of magnitude (4–6) However, higher abundances of AOA have been found in plants treating wastewater with lower concentrations of ammonia (5 to 10 mg N liter^−1^) (7–9). The abundance, activity, and overall contribution of comammox Nitrospira to nitrification in municipal WWTPs is still being uncovered (1, 10–14).

Previous studies have used the 16S rRNA gene of AOB for phylogenetic analysis (15, 16), *in situ* identification (17–20), and quantification of AOB (21–24), in both environmental and engineered systems. Also, the *amoA* gene encoding the AMO enzyme has been used widely as process-specific marker gene for the detection, quantification, and classification of AOB. However, analyses based on sequencing and quantifying DNA or rRNA gene copy numbers provide only limited information on cell activity. Furthermore, it has been shown that the abundance of AOB and AOA in activated sludge does often not correlate with observed rates of ammonia removal (25, 26). Aoi et al. (27) showed that *amoA* mRNA shows sensitive response to ammonia oxidation activity and that quantitative PCR on *amoA* cDNA can be used as a process-specific biomarker of ammonia oxidation in activated sludge.

It has been previously reported that nitrification by AOB is dependent upon different environmental factors, such as dissolved oxygen concentrations (28–30), pH (29, 31), and temperature (29, 30). Many WWTPs in temperate climate zones experience a decline in nitrification performance when wastewater temperatures drop below 15°C (32), although psychrophilic nitrification has been shown to occur in wetland sediments and wastewater at temperatures as low as 5°C (33, 34). Because nitrification represents a substantial challenge for wastewater treatment facilities in cold climates, effluent ammonia concentrations are for practical reasons often not regulated (3).

While ample information is available on the microbial community composition of activated sludge systems (35–39), little information is available on the microbial composition and activities of sludges in full-scale WWTPs that lose their nitrification activity at cold temperatures. Therefore, a better understanding of the structure and function of nitrifying sludge communities in WWTPs experiencing cold temperature nitrification failure may be essential for improving and optimizing wastewater treatment processes at cold temperatures.

The objective of this study was to explore the relationship between community structure and transcriptional activity of ammonia-oxidizing bacteria during cold temperature nitrification failure in three parallel full-scale sequencing batch reactors (SBRs) treating municipal wastewater. We used DNA and cDNA amplicon sequencing and quantitative PCR to identify and quantify 16S rRNA, *amoA* genes, and transcripts in DNA and RNA extracts from activated sludge samples. Sludge samples were collected during summer and fall (the warm wastewater seasons) and when wastewater temperatures were below 15°C (winter and spring) from a WWTP in the Upper Midwest of the United States. By resolving seasonal changes in AOB abundance and species-specific fluctuations in *amoA* gene expression, we aim to identify which ammonia-oxidizing bacteria are the most active populations when water temperatures are low. The study contributes to a better understanding of the composition and activity of AOB communities in nitrifying SBRs experiencing seasonal fluctuations in nitrification performance.

## RESULTS

### Seasonally changing plant parameters and performance.

**FIG 1:**
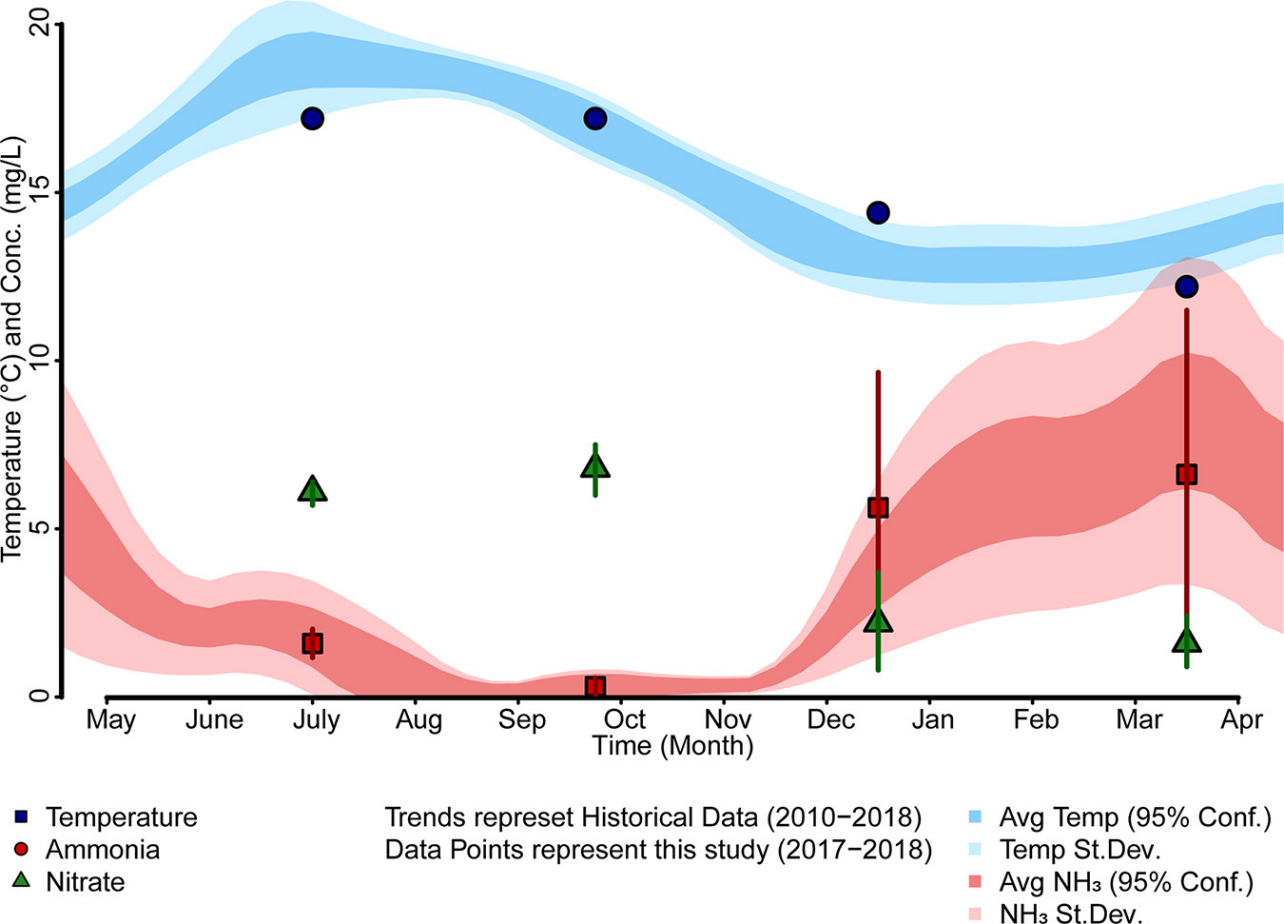
Seasonal variation of water temperature, effluent ammonia, and nitrate concentrations at wastewater treatment plants (WWTPs) in Northern Minnesota, USA, between 2010 and 2018. Average effluent ammonia concentrations between 2010 and 2018 are shown in shades of red, with the average (95% confidence interval [CI]) in dark red and the standard deviation in light red. The average (95% CI) influent wastewater temperature is shown in dark blue with the standard deviation in light blue. The individual data points are the influent water temperature (blue circles), effluent ammonia concentration (red squares), and nitrate (green triangles) with standard deviations during the sampling period of this study.

Historical data records (2010 to 2018) provided by the wastewater treatment plant were used to plot the average annual influent water temperature and effluent ammonia concentrations (Fig. 1). Other plant operational parameters and performance metrics for the sampling period can be found in Table S1 in the supplemental material (40). Influent water temperature and effluent ammonia concentrations revealed an inverse relationship, with lower temperatures in winter and spring correlating with raising ammonia concentrations in the plant effluent. At the wastewater treatment facility in Northern Minnesota where we studied in this project, as well as other wastewater treatment plants in continental climate zones, the annual decrease in plant ammonia removal performance is common and generally referred to as cold temperature nitrification failure. In Fig. 1, ammonia and nitrate concentrations in the effluent during our sampling events in summer, fall, and winter of 2017 and spring of 2018 are shown as individual data points with standard deviations. The average effluent ammonia concentrations during our seasonal sampling trips ranged from 1.59 ± 0.43 mg/liter in the summer, 0.31 ± 0.26 mg/liter in the fall, and 5.63 ± 4.02 mg/liter in the winter of 2017 to 6.62 ± 4.88 mg/liter in the spring of 2018. Except for the sampling event in fall 2017, the effluent ammonia concentrations were always within the standard deviation of the annual average effluent ammonia concentrations recorded from 2010 to 2018 (Fig. 1, red-shaded area) for the three sequencing batch reactors.

**FIG 2:**
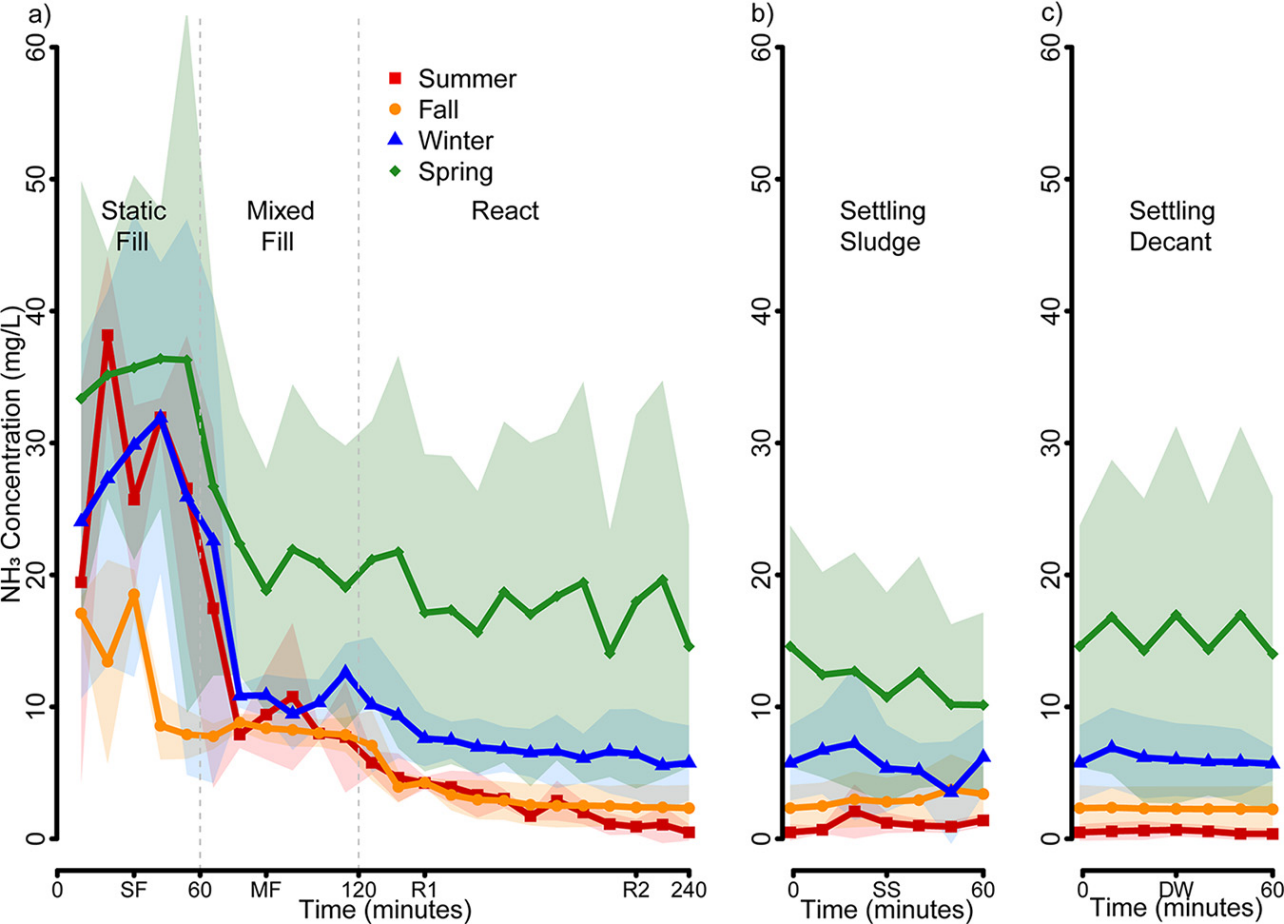
Seasonal variation of average ammonia concentrations (mg/liter) over the duration of a 5-h reaction cycle in three parallel sequencing batch reactors. Average ammonia concentration for summer 2017 (red squares), fall 2017 (orange circles), winter 2017 (blue triangles), and spring 2018 (green diamonds). Standard deviations for each season are shown as shaded areas of the respective season color. (a) Average ammonia concentrations during the first 240 min of a complete reaction cycle. Each cycle starts with the static fill (SF) phase (0 to 60 min) and then goes on to a mixed fill phase (60 to 120 min), followed by two aerobic reaction phases (R1 + R2, 120 to 240 min), during which the sludge is aerated (R1) and mechanically mixed (R2). (b) Average ammonia concentrations in the settled sludge during a 60-min settling phase (SS) following phase R2. (c) Average ammonia concentrations in the decanted water (effluent supernatant) (DW) above the settled sludge during a 60 min settling phase following phase R2. The data in panels b and c were recorded simultaneously from samples collected from the bottom (settled sludge) and top (decanted effluent water) of each sequencing batch reaction during the 60-min settling phase. Individual reactor data are provided in Fig. S1 in the supplemental material.

Fig. 2 shows how fast and to what extent the influent ammonia is removed over a full reactor cycle during each of the four seasonal sampling events. Inflow ammonia concentrations during the static fill (SF) phase fluctuated strongly, with an average of 25 ± 14 mg/liter, probably due to seasonal and daily variations in water flow. Nitrate concentrations are shown throughout the full reactor cycle in Fig. S2 of the supplemental material.

Once the reactors mix influent wastewater with activated sludge during the mixed fill (MF) phase, the average ammonia concentrations quickly decreased to 9.0 ± 2.7 mg/liter but only in the summer, fall, and winter. Ammonia concentration experienced a statistically significant decline during summer, fall, and winter (analysis of variance [ANOVA]: summer *P* < 2.47e−7, fall *P* < 5.00e−4, and winter *P* < 1.14e−4). In the spring MF phase, ammonia concentrations decreased to only 20 ± 9 mg/liter, which was significantly higher than what we quantified for the other three seasons and but did not experience any further statistically significant biodegradation (ANOVA: spring *P* = 0.094). During the aerobic react cycle, the ammonia concentrations further decreased to 0.5 mg/liter in summer, 2.3 mg/liter in fall, and 5.8 mg/liter in winter. In the spring, the ammonia concentrations decreased to 14.6 mg/liter at the end of the aerobic react cycle, which was again significantly higher than what we quantified at the end of the react cycle for the other three seasons (*t* test: summer-spring *P* = 0.0025, fall-spring *P* = 0.0044, and winter-spring *P* = 0.0175).

We did not observe any statistical difference between summer and fall ammonia concentrations during the aerobic react cycle and settling phase. The ammonia concentrations during winter and spring were always higher than summer and fall ammonia concentrations but were also significantly different from each other. Seasonal loss of nitrifications performance was most obvious during the spring, resulting in high ammonia effluent concentrations but also high standard deviations among the three reactors due to diurnal loading. During the anaerobic settling phase, we quantified ammonia concentrations in the settled sludge (Fig. 2b) and in the decanted water (Fig. 2c), but these samples did not show a statistically significant further removal of ammonia for any season (*t* test: summer *P* = 0.7498, fall *P* = 0.4852, winter 0.3612, and spring = 0.5502).

### Seasonal variation in *amoA* gene and transcript abundance.

**FIG 3:**
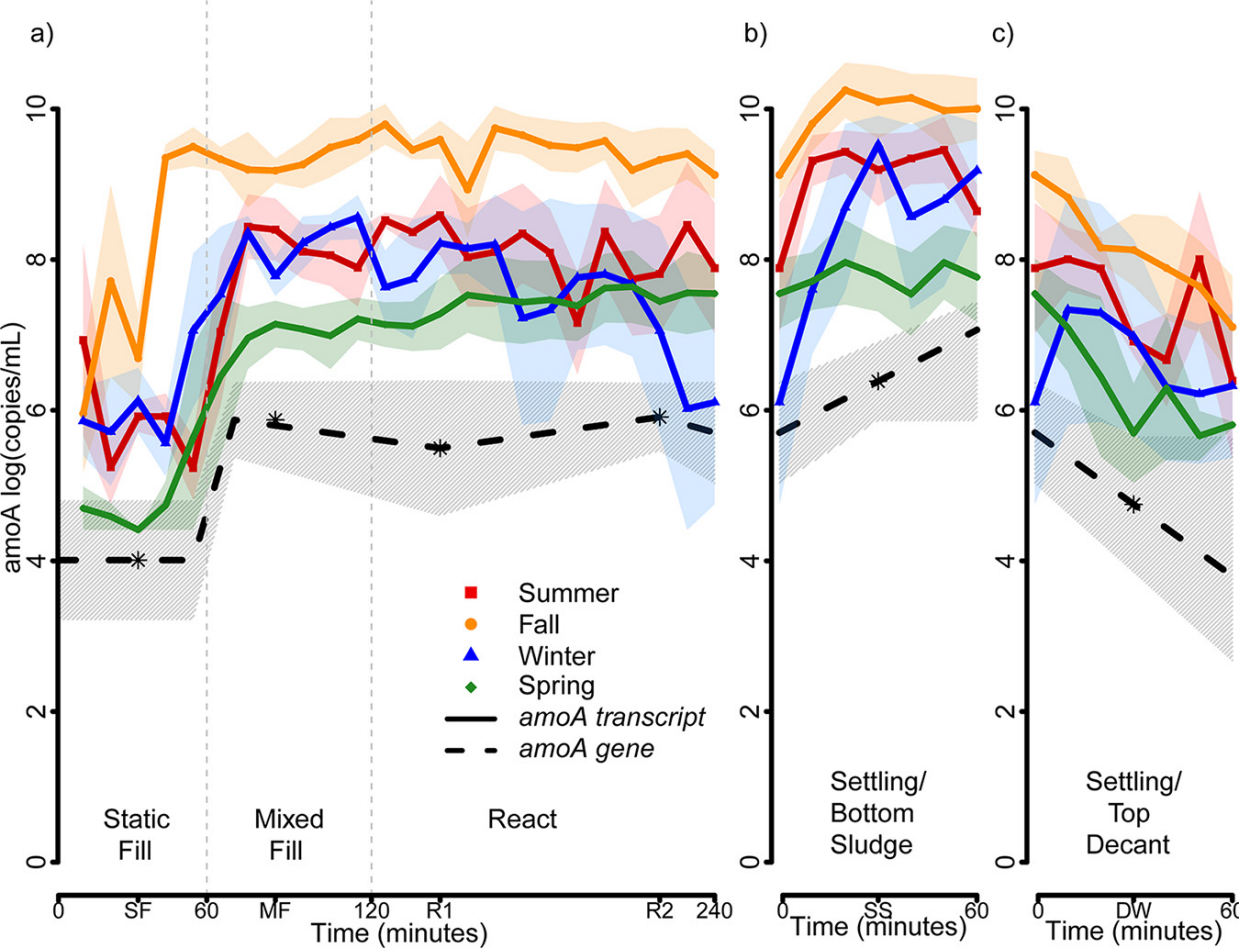
Seasonal variation of *amoA* transcript and gene copy numbers over of a 5-h reaction cycle in three parallel sequencing batch reactors. Transcript and gene copy numbers were quantified in 10-min intervals. (a) *amoA* transcript and gene copy numbers during the first 240 min of a complete reaction cycle. Each cycle starts with the static fill (SF) phase (0 to 60 min) and then goes on to a mixed fill phase (60 to 120 min), followed by two aerobic reaction phases (R1 + R2, 120 to 240 min), during which the sludge is aerated (R1) and mechanically mixed (R2). (b) *amoA* transcript and gene copy numbers in the settled sludge during a 60-min settling phase (SS) following phase R2. (c) *amoA* transcript and gene copy numbers in the decanted water (effluent supernatant) (DW) above the settled sludge during a 60-min settling phase following phase R2. The data in panels b and c were recorded simultaneously from samples collected from the bottom (settled sludge) and top (decanted effluent water) of each sequencing batch reaction during the 60-min settling phase. In all three panels, *amoA* transcript copy numbers (solid lines) are shown for summer 2017 (red squares), fall 2017 (orange circles), winter 2017 (blue triangles), and spring 2018 (green diamonds). Standard deviations for each season are shown as shaded areas of the respective season color. The black dashed line and gray hatched area represent the annual average *amoA* gene copy numbers for all four seasons.

We quantified *amoA* transcript copy numbers at 10-minute intervals throughout complete reactor cycles of all three sequencing batch reactors in each of the four seasons (Fig. 3). *amoA* transcript copy numbers were highly variable during the static fill phase for all seasons, averaging 6.1 ± 1.7 log(copies/mL). The onset of mechanical mixing (MF) resulted in a significant increase in *amoA* transcript copy numbers in all four seasons. The highest abundance of *amoA* transcript copies were quantified in the fall with an average of 9.4 ± 0.5 log(copies/mL) throughout the MF and aerobic react cycle phases (R1 and R2). Fall *amoA* transcript copy numbers were significantly higher during MF, R1, and R2 compared to the three other seasons (*P* ≪ 0.05; refer to Table S2 in the supplemental material for a complete tabulation). The *amoA* transcript copy numbers in summer and winter were not statistically different (*P* = 0.09), with on average 8.1 ± 0.9 log(copies/mL) in the summer and 7.7 ± 1.5 log(copies/mL) in the winter. While *amoA* transcript copy numbers in winter and spring were not significantly different either (*P* = 0.08), spring *amoA* transcript copy numbers were with an average of 7.3 ± 0.8 log(copies/mL) significantly lower than summer transcript copy numbers (*P* = 6.3e−6).

The *amoA* transcript copy numbers significantly increased during the settling phase as activated sludge biomass sank to the bottom of the reactor (SS). Average log(copies/mL) of *amoA* transcripts were 9.2 ± 0.6 in summer, 10.0 ± 0.6 in fall, 8.7 ± 1.5 in winter, and 7.8 ± 0.8 in the spring. Respectively, we observed a decrease in *amoA* transcript copy numbers in the decanted water (DW) as the suspended solids settled. The decanted water averaged log(copies/mL) of *amoA* transcripts ranging from 7.3 ± 1.2 in the summer, 8.0 ± 1.0 in fall, 7.7 ± 1.8 in winter, and 6.2 ± 1.1 in spring.

Quantification of *amoA* gene copy numbers did not reveal statistically significant variations throughout reactor cycles SF, MF, R1, and R2 for the four seasons. The black dashed line in the graph shown in Fig. 3 represents the average *amoA* gene copy number for all four seasons, while the gray-shaded area represents the seasonal variability as standard deviation. The average SF *amoA* gene abundance was 4.0 ± 0.8 log(copies/mL). The average *amoA* gene abundance throughout MF, R1, and R2 was 5.8 ± 0.7 log(copies/mL). During the settling phase the *amoA* gene copy numbers increased to 6.4 ± 0.5 log(copies/mL) in the bottom sludge (SS) and decreased to 4.8 ± 0.9 log(copies/mL) in the decanted water above.

Beyond ammonia-oxidizing bacteria, we also quantified *amoA* genes and transcripts of ammonia-oxidizing archaea (AOA). The average concentration of AOA *amoA* genes in the activated sludge was 3.5 ± 0.5 log(copies/mL), which was about 2.5 orders of magnitude lower than average AOB amoA gene copy numbers. A summary of the quantified average AOA *amoA* genes copy numbers for all fours seasons is shown in Fig. S3 in the supplemental material. In 80% of the analyzed samples, AOA *amoA* transcripts were below the quantitative PCR (qPCR) assay detection limit of 4 log(transcript copies/mL).

### Ammonia monooxygenase (*amoA*) gene and transcript sequencing.

**FIG 4:**
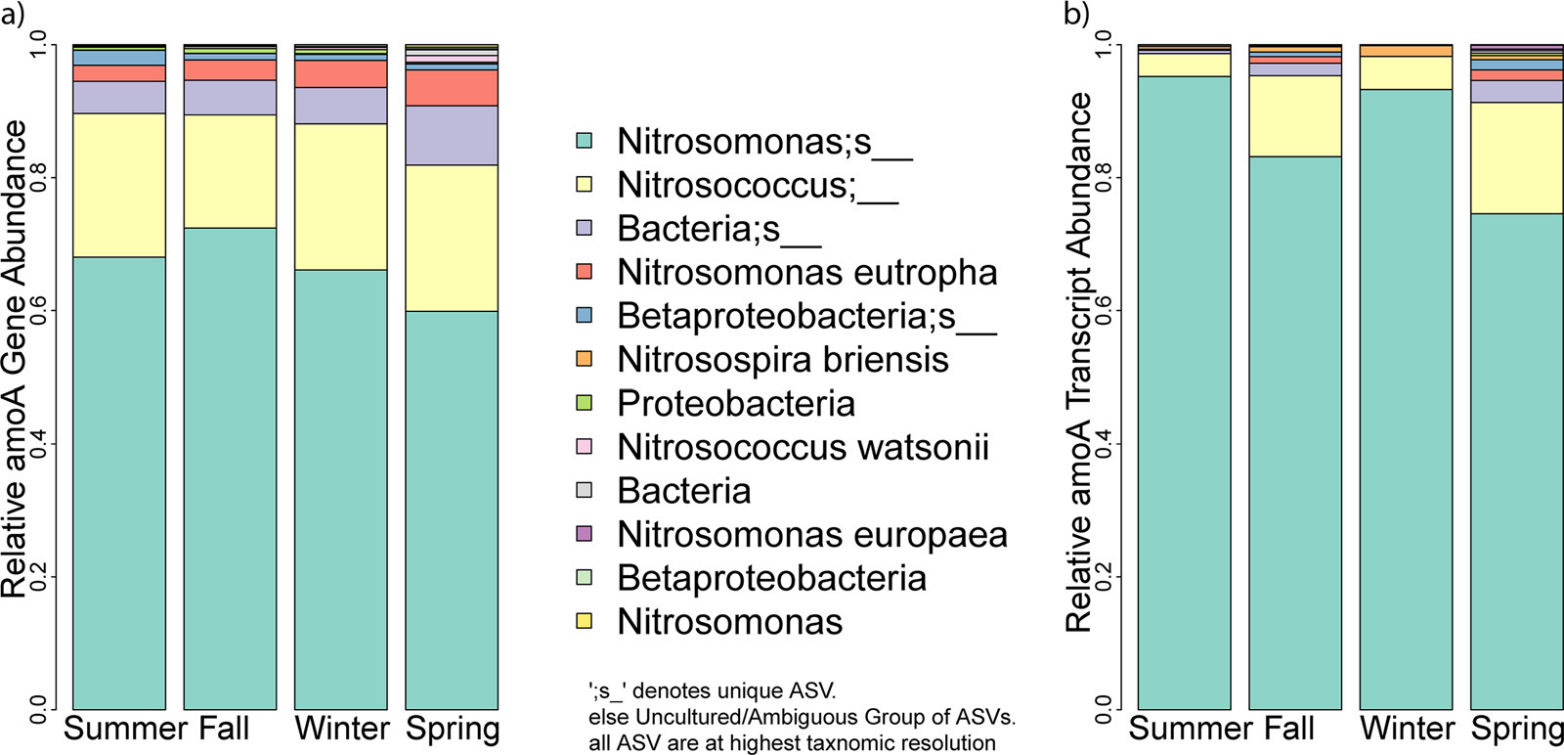
Seasonal variation in average taxonomic composition of *amoA* genes and transcript within three parallel SBRs. (a) *amoA* gene-based ammonia-oxidizing bacteria community composition. (b) *amoA* transcript-based ammonia-oxidizing bacteria community composition. In the middle between panels a and b, each classified *amoA* amplicon sequence variant (ASV) cluster is shown in a different color and listed by its taxonomic name and relative sequence abundance starting with the most abundant taxa on top. ASVs were assigned the highest taxonomic level possible. Single classified ASVs are denoted by an underscore at the end. ASVs that shared an identical taxonomic assignment were grouped and are listed without the underscore at the end. Fig. S3 in the supplemental material shows how each season’s *amoA* gene and transcript composition shifts over a complete sequencing batch reactor (SBR) reaction cycle, comparing the phases static fill, mixed fill, aerobic react with (R1) and without (R2) mechanical mixing, and the *amoA* gene and transcript composition in the settled sludge and in the decanted water.

Sequencing the *amoA* gene and transcript revealed taxonomic shifts in the community composition of ammonia-oxidizing bacteria (AOB) throughout the reactor cycle. Based on the Faith Phylogenetic Diversity Index (41), the AOB *amoA* gene community in the influent wastewater (SF) had a higher diversity than the observed *amoA* gene diversity in the activated sludge after mixing (Fig. S4 in the supplemental material). Overall *amoA* gene and transcript diversity was relatively low. Only five amplicon sequence variants (ASVs) had an average relative abundance greater than 1% throughout the whole year. These five ASVs accounted for >97.6% of all *amoA* gene sequences and >98.6% of all *amoA* transcript sequences (Fig. 4). During static fill (SF), when primarily domestic wastewater was pumped into the SBRs, AOB *amoA* gene diversity was dominated by two ASVs, namely, a Nitrosococcus sp. with 43.75% ± 8.23% relative *amoA* sequence abundance and an uncultured Nitrosomonas sp. with 32.48% ± 12.18% relative *amoA* sequence abundance. Among the sequenced *amoA* transcripts, these two taxa made up 26.22% ± 5.92% and 59.30% ± 4.11% of the relative sequence abundance, respectively. Once the influent wastewater got mixed in with the activated sludge, the relative sequence abundance of the uncultured Nitrosomonas sp. increased to 66.61% ± 7.4% for the *amoA* gene and 86.56% ± 9.42% for *amoA* transcripts. The relative sequence abundance of the Nitrosococcus sp. ASV cluster, on the other hand, decreased to 20.66% ± 4.25% for *amoA* genes and 9.34% ± 6.76% for *amoA* transcripts (Fig. 4). Fig. S5 shows a breakdown of the *amoA* gene and transcript composition for each season. There were no major differences in *amoA* gene and transcript composition among seasons (Fig. S5 in the supplemental material). Single, double, and low-abundance sequence reads (<0.01%) were excluded from the analysis due to lack of statistical significance. Low-abundance reads were predominantly associated with the low biomass samples taken during static fill and decanted water, which had overall sequencing read numbers of <10,000 reads/sample. For the rest of the data, sequence read abundances of the three individual reactors were averaged at each respective sampling time period.

**FIG 5:**
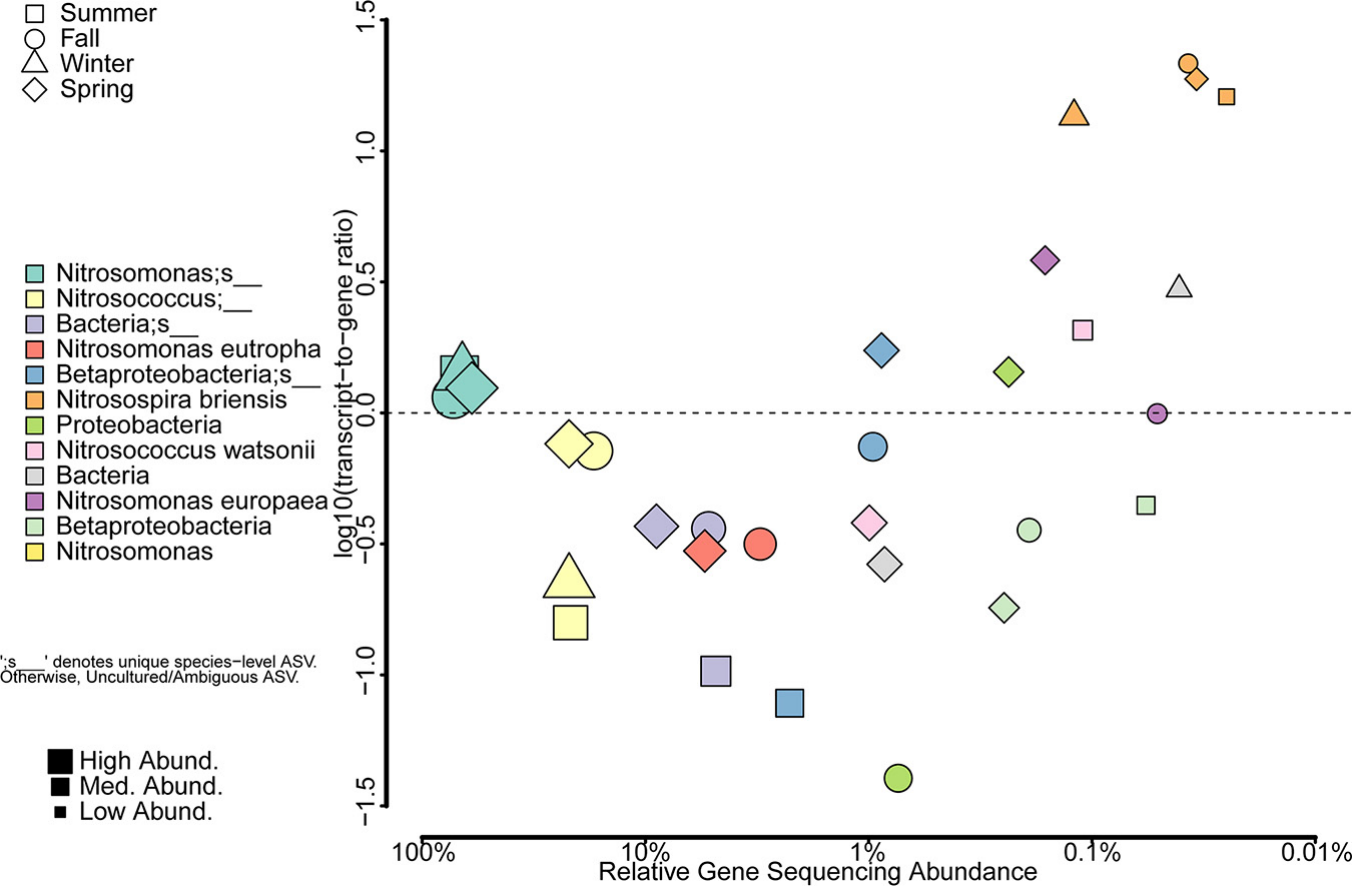
Log gene abundance versus log transcript-to-gene ratio for classified *amoA* amplicon sequence variants. ASVs were classified at the lowest taxonomic level possible and ranked by their relative gene abundance to the left. Individual ASVs are denoted by an underscore at the end of their taxa description. All other listed *amoA* taxa represent multiple ASV with an identical taxonomic assignment. Each point is the mean of the triplicate reactors, which showed little variation, while the shape and outline color of each symbol differentiate between sampling seasons. Red squares, summer; orange circles, fall; blue triangles, winter; green diamonds, spring. Symbol size represents the relative gene abundance of the respective *amoA* taxa.

We normalized transcript copy numbers to *amoA* gene relative sequence abundance for each identified ASV cluster to discern differences in *amoA* gene expression activity among the different taxa of ammonia-oxidizing bacteria that we identified in the activated sludge samples during the four seasons. In Fig. 5, we plotted the relative *amoA* gene abundance of each ASV on the *x* axis and the log-transformed ratio of the relative abundances of *amoA* transcripts-to-genes on the y-axis. The graph in Fig. 5 shows that the uncultured Nitrosomonas sp. ASV cluster showing the highest *amoA* gene and transcript relative sequence abundance during the four seasons was not the most active with respect to *amoA* transcripts expressed per *amoA* gene observed. According to Fig. 5, an ASV related to Nitrosospira briensis had a 10-fold higher *amoA* transcript-to-gene ratio than the uncultured Nitrosomonas sp. ASV. While N. briensis had a relative *amoA* gene sequence abundance of only 0.05% ± 0.04%, its high transcript-to-gene ratio suggests either elevated *amoA* gene expression activity or extended *amoA* transcript half-life during the aerobic reaction cycle in the studied SBRs. However, it was a general trend in the normalized gene expression data that higher abundance ASVs had a transcript-to-gene ratio closer to 1:1, while the ASV with the lower relative abundance of *amoA* genes appeared to be the most “active” based on *amoA* transcripts per *amoA* gene.

**FIG 6:**
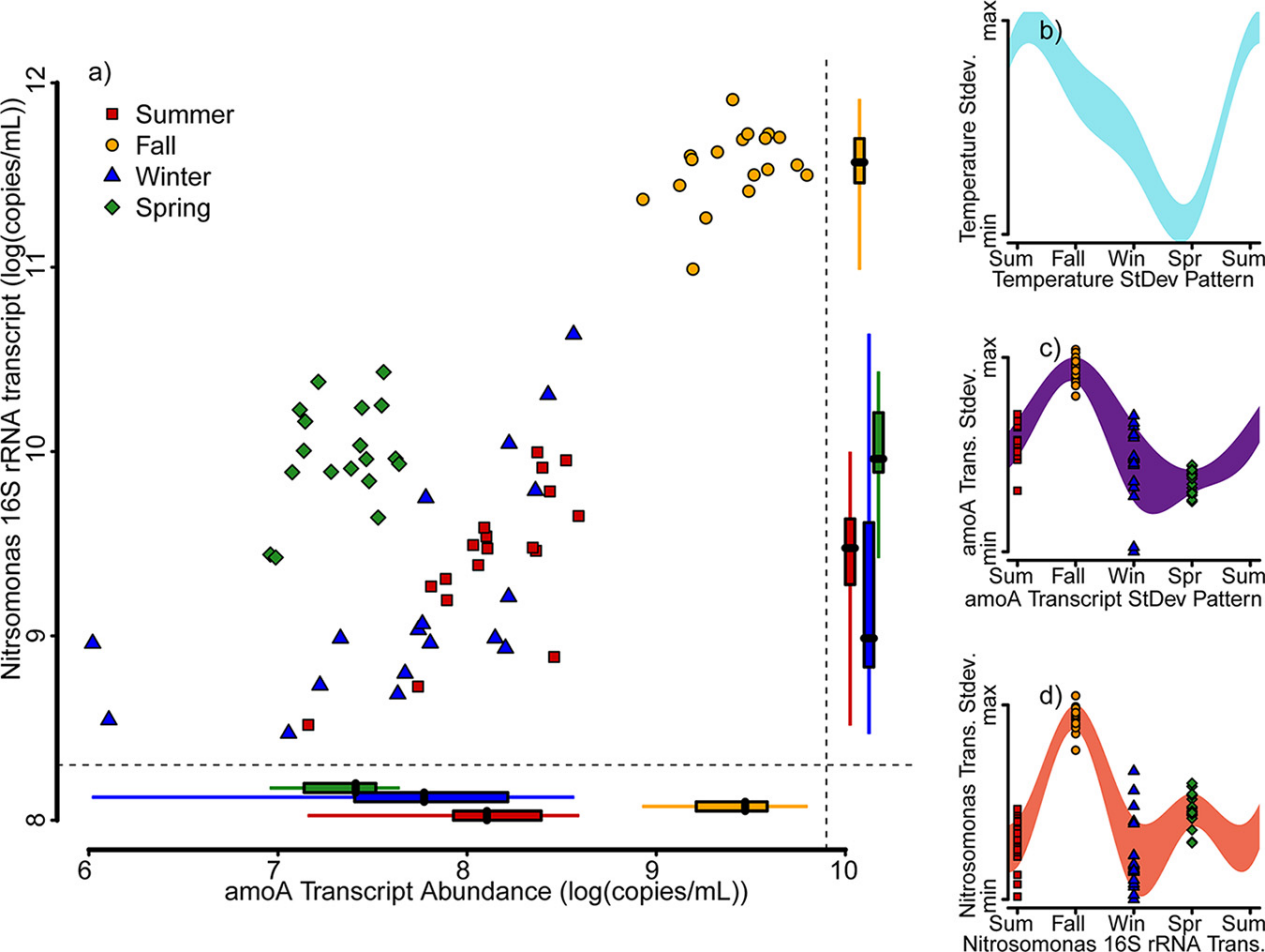
Seasonal correlation of total *amoA* transcript copy numbers and Nitrosomonas 16S rRNA gene transcript copy numbers. (a) *amoA* transcript copy numbers (*x* axis) and Nitrosomonas 16S rRNA gene transcript copy numbers (*y* axis). Summer samples are shown as red squares, fall samples are orange circles, winter samples are blue triangles, and spring samples are green diamonds. Box-and-whisker plots on the *x* and *y* axes show median and 25% to 75% interquartile ranges of the seasonal transcript abundance data. (b to d) Standard deviations of monthly changes in temperature (b), *amoA* transcript copy numbers (c), and Nitrosomonas 16S rRNA gene transcript copy numbers (d). Panels c and d also show the data from individual samples collected during the respective seasons (same data as in panel a). The data to the left of the red dashed line is the experimental data measured in this study. The data to the right of the dashed line repeats the measured data pattern for two consecutive years (no real data) to emphasize the seasonal variation and correlative trends in water temperature, *amoA* transcripts, and Nitrosomonas 16S rRNA transcript copy numbers.

In order to gain further insights into seasonal variations in ammonia oxidizer gene expression activity during the aerobic react cycle (R1 and R2) of the sequencing batch reactors, we decided to take a closer look at the taxon Nitrosomonas because it was the most abundant nitrifier taxa that also consistently showed expression of the *amoA* gene throughout all phases of a complete reaction cycle in all four seasons. In a previous study, we had already obtained seasonal 16S rRNA transcript sequence data, as well as quantitative information on the abundance of Nitrosomonas spp. 16S rRNA transcript copy numbers of activated sludge samples from the same plant (40). We used these data together with the *amoA* transcript reverse transcription (RT)-qPCR data obtained in this study to plot the graph shown in Fig. 6. In Fig. 6, the relative abundance of Nitrosomonas spp. 16S rRNA transcripts was multiplied by the total 16S rRNA transcript copy number per mL and plotted against *amoA* transcript copy numbers per mL for all seasonal samples. The Tukey box and whisker graphs at the bottom and right side of Fig. 6 show the range of seasonal data of *amoA* and Nitrosomonas spp. 16S rRNA transcript abundances.

The *amoA* transcript abundances were the highest in the fall (9.4 ± 0.2 log[transcripts/mL]) followed by summer (8.1 ± 0.4 log[transcripts/mL]) and then winter (7.7 ± 0.7 log[transcripts/mL]) with the lowest in the spring (7.3 ± 0.2 log[transcripts/mL]). Winter and spring did not show statistically different average transcript abundances (*P* > 0.065). The seasonal trend of *amoA* transcript abundance correlated well with the seasonal variation in inflow wastewater temperature as shown in Fig. 6b and c. However, Nitrosomonas spp. 16S rRNA transcript abundance dynamics did not follow the same seasonal trend as that shown in Fig. 6d. Nitrosomonas spp. 16S rRNA transcript copy numbers were highest in the fall (11.5 ± 0.2 log[transcripts/mL]), followed by spring (10.0 ± 0.3 log[transcripts/mL]), summer (9.4 ± 0.4 log[transcripts/mL]), and winter (9.2 ± 0.6 log[transcripts/mL]). Winter and summer were statistically similar (*P* > 0.246), while spring and fall were significantly different from each other (*P* < 0.05). A linear regression of influent wastewater temperature throughout the seasons with the log-normalized *amoA* and Nitrosomonas 16S rRNA transcript copy numbers is shown in Fig. S6 of the supplemental material. In Fig. 1, we showed the seasonal dynamics and inverse correlation of water temperature with effluent ammonia concentrations. By comparing Fig. 6b and c, it becomes apparent that water temperature seems to also affect *amoA* gene expression, providing a potential explanation for the observed decrease in ammonia removal with a decline in water temperature. However, Nitrosomonas spp. 16S rRNA transcripts do not appear to follow the same dynamic trend, further providing evidence that Nitrosomonas sp. abundance is not a reliable measure of reactor ammonia oxidation performance.

## DISCUSSION

### Seasonal nitrification failure.

Seasonal nitrification failure did occur during the sampling year, with the average ammonia concentration in the DW rising to 6.2 ± 2.8 mg/liter in winter and 10.1 ± 7.0 mg/liter in spring. The ammonia concentration had large standard deviations in spring, likely due to differences in inflow concentrations of each reactor during the filling processes (SF and MF). For each sample trip, the start times for filling a reactor were at approximately 10 a.m., 12 p.m., and 2 p.m. Whichever reactor started filling closest to 10 a.m. typically reported a lower concentration of ammonia in the influent wastewater. This is a result of the differences in the diurnal volumetric flows coming into the treatment plant, where early mornings have an increased use of showers and running water, which creates a peak volume of influent wastewater but lowers the concentration of ammonia (42). This creates different initial concentrations of ammonia in the three parallel reactors, while the actual removal of ammonia in each reactor does not show statistically significant differences (ANOVA: summer *P* = 0.716, fall *P* = 0.655, winter *P* = 0.231, and spring *P* = 0.773) as shown in Fig. S7 of the supplemental material.

The full-scale sequencing batch reactors in this study typically perform simultaneous nitrification, denitrification, and phosphorous removal. Similar lab-scale reactors have sustained nitrification, denitrification, and phosphorous removal at temperatures as low as 10°C but have prolonged aeration periods of 4 h (43). Aeration already accounts for approximately 44% of the operational costs at wastewater treatment facilities like the one sampled in this study. Extending the period of aeration will be a significant cost factor for municipalities (44).

### Seasonal dynamics of nitrifier abundance and activity.

Based on qPCR experiments on reactor DNA extractions, our results show that *amoA* gene copy numbers were stable throughout the year. We observed 5.8 ± 0.7 log(copies/mL) of *amoA* genes, while similar studies have shown between 5.5 to 7.5 log(copies/mL) of *amoA* genes for ammonia-oxidizing bacteria in several municipal and industrial wastewater treatment facilities (32, 45, 46). However, following RNA extraction and cDNA synthesis, we observed a strong correlation of *amoA* transcript copy numbers with wastewater temperature. According to the observed trends in wastewater temperature and nitrification performance (Fig. 1), *amoA* transcript abundance was highest in the fall following a prolonged period of warm summer temperatures (Fig. 3). The *amoA* transcript abundance was lowest in the spring after months of cold winter temperatures (Fig. 2). This is consistent with previously reported observations of the seasonal dynamics in cell (47) and transcript (48–50) abundance among nitrifying populations and likely explains why wastewater treatment plants experience seasonal nitrification failure. Interesting to us was that we observed that the 16S rRNA transcript abundance of Nitrosomonas spp. did not decrease with decreasing wastewater temperatures. While we quantified the highest abundance of Nitrosomonas spp. 16S rRNA transcripts in the fall, the second highest transcript abundances were recorded in spring. This means that Nitrosomonas spp. increased their average protein synthesis potential in the spring to a level higher than the observed average protein synthesis potentials quantified for winter and summer (Fig. 6). This suggests that Nitrosomonas spp. can maintain their ribosome numbers at low wastewater temperatures, once the temperature stabilizes. If an increase in ribosome counts is indicative of cell metabolic activity and growth, then it appears that the observed increase in protein synthesis potential in Nitrosomonas spp. during the spring is not associated with ammonia oxidation since ammonia concentrations in the effluent are highest during in spring. Also, Nitrosomonas spp. cells abundance (16S rRNA gene copy numbers) does not decrease in spring, raising the question of how Nitrosomonas spp. maintains cell growth based on an energy metabolism other than ammonia oxidation. Further work is required to analyze metabolic flexibility and potential phenotypic adaptation of growth and energy metabolism in Nitrosomonas spp. induced by seasonal variations in wastewater temperature and plant operational conditions (aeration) in full-scale sequencing batch reactions.

Maintaining a constant cell abundance in an SBR requires consistent population growth to avoid washout and a decline in cell numbers. If the growth of Nitrosomonas spp. during the cold season is not associated with a measurable extent of ammonia oxidization, it might indicate that Nitrosomonas spp. can grow on a minimal amount of energy from ammonia oxidation, or they might switch to an energy metabolism other than ammonia oxidation at lower temperatures in order to be able to continue to grow and maintain a stable population size. Previous studies have demonstrated that Nitrosomonas europaea has both nitrite reductase and nitric-oxide reductase (51), and several species have urease and can grow on urea (52), but whether or not these are predominantly used by Nitrosomonas in activated sludge has not been explored. These pathways are all linked to the nitrogen cycle. Instead of predominantly performing ammonia oxidation, perhaps performing different reactions of the nitrogen cycle as the reactors alternate between aerobic and anaerobic cycles is sufficient for sustained growth. However, ammonia-oxidizing bacteria fix carbon via the Calvin Cycle, for which they have to invest energy in reverse electron flow to generate the reducing equivalents necessary to reduce CO_2_ to the oxidation state of carbon in biomass (53–55). While Nitrospira comammox organisms are not present in our system, recent analysis has shown that their metabolic diversity might possibly be due to various environmental conditions such as oxygen availability (56). Potential alternative sources of energy for Nitrosomonas, e.g., *related to* urea metabolism and/or denitrification, need to be further investigated to determine whether seasonal shifts in gene expression of these pathways might explain the observed stable population abundance of Nitrosomonas sp. during the winter.

We also previously observed that general bacterial 16S rRNA transcript copy numbers significantly declined in the winter once reactors start to get aerated with cold ambient air (40). In this study, the abundance *of amoA* transcript copy numbers slowly declined only in response to the onset of aeration in the winter. This suggests that while the overall protein synthesis potential of the activated sludge microbial community has immediate responses, transcription of *amoA* genes only progressively declines and is less impaired by a decline in reactor water temperature during cold air aeration. While we observed a correlation between the seasonal abundance of *amoA* transcripts and reactor nitrification performance, the decrease in community 16S rRNA transcripts at the onset of aeration is indicative of a general decline in microbial activity in the activated sludge during cold season reaction cycles. This suggests that the decrease in ammonia removal at colder temperatures is not solely associated with the abundance and activity of uncultured Nitrosomonas spp. but also associated with the activity of other activated sludge community members that might directly or indirectly affect ammonia removal from wastewater. The consistent *amoA* transcript copy numbers during the transition from the “mixed fill” to the “aeration” phase is supported by previously reported findings that *amoA* expression is controlled and regulated by the presence and concentration of ammonium not oxygen (57).

### Sequence diversity of *amoA* genes.

Overall, we observed little seasonal variation in *amoA* gene sequence diversity and no significant differences between the ammonia monooxygenase gene community composition of the triplicate sequencing batch reactors. The lack of seasonal variations in ammonia oxidizer populations has been previously reported in 12 wastewater treatment plants near Tokyo (58); however, these treatment plants were in a subtropical climate where their reported ambient air temperatures varied between 14°C to 31°C, whereas the WWTP under investigation in this study experiences ambient air temperatures ranging from –34°C to 28°C throughout a year. The vast majority of amoA sequences in the influent wastewater and the activated sludge were related to uncultured Nitrosomonas and Nitrosococcus. In fact, only 4 ASV clusters were related to cultured species accounting for about ~3.5% of *amoA* gene relative sequence abundance and only ~1.6% of the relative *amoA* transcript abundance. This is reflective of the “Great Plate Counting Anomaly (59)” and shows that the majority of available reference genes is dependent on the few cultured organisms and that the majority of environmental sequences comes from uncultured species. Also, Wang et al. (60) predominantly identified unknown Nitrosomonas species in a terminal restriction fragment-length polymorphism (T-RFLP) fingerprinting study comparing different wastewater treatment plants. However, they did not report on the presence of any Nitrosococcus sequences, although they used the same *amoA* gene forward primer and a reverse primer only slightly different from that used in this study. Additionally, neither of our previous 16S rRNA amplicon sequencing studies (32) detected Nitrosococcus, which might be a limitation in the 16S rRNA primer selection (61). However, the identified Nitrosococcus *amoA* sequences are very similar to a N. europaea Nm50 *amoA* gene cluster, and potential horizontal gene transfer between these lineages has previously been discussed (19).

### Abundance of *amoA* transcripts.

We calculated the ratio of the relative abundance of *amoA* transcripts to *amoA* genes for each *amoA* gene ASV to normalize and compare nitrifier ammonia oxidation activity. Based on the Faiths phylogenetic diversity index shown in the supplemental material (Fig. S4), we observed less diversity of nitrifiers expressing *amoA* transcripts than there was actual amoA gene diversity, resulting in most ASV clusters having low transcript-to-gene ratios. The majority of *amoA* transcripts and *amoA* gene sequences that we identified belong to an uncultured group of Nitrosomonas sp.; however, the ratio of transcripts to1 genes was only slightly greater than 1 (1.3 ± 0.1 *amoA* transcripts per *amoA* gene). The most transcriptionally “active” *amoA* gene ASV across all four seasons was N. briensis, which averaged 17.5 ± 3.5 transcripts/genes. Bollmann et al. (62) have previously shown that N. briensis is capable of maintaining stable biomass levels while *amoA gene* transcription slowly decreased over time in ammonia starvation experiments. N. briensis was first isolated by Helene Winogradsky and is typically found in freshwater ecosystems, agricultural fields, and meadows (63, 64). It remains to be shown why N. briensis had the highest amoA transcript to gene ratio in our reactors despite its relative low abundance in the activated sludge samples. Species competition or significant environmental pressure (nutrient limitation, predation, phages) might keep species abundance low (65) while individual cells show high transcriptional activity. Further research should explore the role of N. briensis in engineered systems such as WWTPs and whether the observed relatively *amoA* transcriptional activity is actually correlated with high ammonia oxidation rates of this taxa in activated sludge.

### Conclusions.

In this study, we quantified the seasonal dynamics of ammonia monooxygenase gene expression in activated sludge from full-scale sequencing batch reactors. We observed strong seasonal variation in *amoA* transcript abundance that correlated with a loss in nitrification performance when wastewater temperatures dropped below 15°C. *amoA* gene abundance was stable throughout the whole year. An uncultured group of Nitrosomonas spp. was the most abundant *amoA* ASV all the year round, suggesting that these Nitrosomonas group can maintain stable abundances and regulate their maintenance metabolism or possess other means of generating energy for cell growth during colder temperatures when nitrification subsides. An ASV cluster identified as most closed related to *amoA* genes of N. briensis showed the highest gene expression per *amoA* gene. The impact of N. briensis on cold temperature ammonia oxidation in activated sludge systems requires warrants studies. We demonstrated that *amoA* transcript quantification can provide new insights to better understand the seasonal variation of nitrification process performance in WWTP in temperate climate zones. However, we just started to unravel the role and potential importance of low-abundance but highly active nitrifier populations for process performance and stability in activated sludge systems.

## MATERIALS AND METHODS

Many of the methods used in this study have been previously been described (40). Among these procedures and methods, there is a detailed description of sample collection and storage, protocols for DNA and RNA extraction, cDNA synthesis, RT-qPCR, 16S rRNA gene amplicon sequencing, and sequence and statistical analyses. In this article, we provide a brief description of the methods used in this study. For more details on each experimental procedure please refer to the previous article (40).

### Sample collection.

Samples were collected at a WWTP in Northern Minnesota on July 1, 2017; October 3, 2017; December 27, 2017; and March 27, 2018, representative of summer, fall, winter, and spring plant performance according to Johnston et al. (32) and Johnston and Behrens (40). Activated sludge samples were collected every 10 minutes throughout the complete reaction cycles of the three parallel SRBs. Sample collection included the reactor phases static fill (SF), mixed fill (MF), aerobic react (R1 at the onset of aeration and R2 just prior to the end of aeration), as well as settled sludge (SS) and decanted water (DW).

### DNA/RNA extraction.

Initial sample volume was 500 mL from which aliquots in 2-mL microcentrifuge tubes were immediately frozen to approximately –72°C in a dry ice/ethanol bath and stored in –80°C until downstream processing. DNA was extracted using the MP BIO FastDNA spin kit for soil (Santa Ana, CA). RNA extractions were performed using Quick-RNA fecal/soil microbe microprep kit by Zymo Research (Irvine, CA). Residual DNA was digested using the TURBO DNA-free kit from Invitrogen (Carlsbad, CA) followed by reverse transcription with the PrimeScript RT-PCR kit from TaKaRa (Kusatsu, Japan). All nucleic acid extracts were quantified on a Qubit 4 fluorometer by Invitrogen (Carlsbad, CA) using the high-sensitivity fluorometric assays kits for DNA and RNA.

### 16S rRNA gene and transcript amplicon sequencing and analysis.

16S rRNA gene and transcript amplicon sequencing was performed by the University of Minnesota’s Genomic Core using V1 to V3 primers and the reaction conditions previously described by Johnston et al. (32). Sequence analysis was performed using DADA2 (41) in RStudio (66) referencing the SILVA rRNA SSU 132 database (67). Statistical analysis was done in RStudio using the package *vegan* (68).

### Amplification and sequencing of *amoA* genes and transcripts.

The primers used for *amoA* gene and transcript amplification and quantification of ammonia-oxidizing bacteria by qPCR and RT-qPCR were amoA-1Fmod (5′-CTGGGGTTTCTACTGGTGGTC-3′) and GenAOBR (5′-GCAGTGATCATCCAGTTGCG-3′) published by Meinhardt et al. in 2015 (69). Additionally, we used the primers GenAOAF (5′-ATAGAGCCTCAAGTAGGAAAGTTCTA-3′) and GenAOAR (5′-CCAAGCGGCCATCCAGCTGTATGTCC-3′) for the detection and quantification of ammonia-oxidizing archaea by qPCR and RT-qPCR (69). For AOB *amoA* gene and transcript sequencing, we used the same forward primer (amoA-1Fmod) but replaced the reverse primer with amoA-2R (5′-CCCCTCKGSAAAGCCTTCTTC-3′) (70), which allowed for slightly longer sequence reads (approximately 490 bp). Sequencing primers were fitted with Nextera adapters. Library preparation and sequencing was performed by the University of Minnesota’s Genomics core facility on a Illumina MiSeq system. Sequencing returned a total of 4,597,063 raw reads of *amoA* gene amplicons and 3,647,505 raw reads of *amoA* transcript amplicons (distribution shown in Fig. S1 in the supplemental material).

Demultiplexed raw sequences were imported into QIIME 2 (version 2019.1; https://qiime2.org/) for initial processing and quality control (71). The DADA2 plugin was used to remove primer sequenced and trim off low quality regions (truncQ = 2) of forward and reverse reads at 290 and 260 bp (72), respectively. The chimera removal and dereplication were also performed in DADA2 using default settings. DADA2 generated amplicon sequence variants (ASVs) were used to generate sequence alignments and masks using the align-to-tree-mafft-fastree pipeline in the q2-phylogeny plugin. The *amoA* gene samples averaged 16,978 ± 6,763 merged read pairs per sample, and the *amoA* transcript samples averaged 13,106 ± 7,431 merged read pairs per sample (distribution shown in Fig. S1 in the supplemental material). For α- and β-diversity estimation, sequence libraries of *amoA* gene and transcripts were rarefied to a sampling depth of 5,000 and 2,000 sequences, respectively.

A custom *amoA* reference sequence database was created for classification and taxonomic analysis of *amoA* sequence reads. For that, high-quality *amoA* sequences with BLAST scores of >430, Hidden Markov model coverage of >80%, and read length of at least 200 amino acids were downloaded from the FunGene database (http://fungene.cme.msu.edu/) (73). The *amoA* database sequences were trimmed to match the read length of our *amoA* sequencing primers. Database sequences were classified through Entrez using a custom Perl script and the NCBI taxonomy. A Naïve Bayes classifier was then trained using the database sequences and their taxonomic annotation in order to classify the *amoA* gene and transcript sequences obtained in this study using the q2-feature-classifier plugin.

### Ammonia and nitrate quantification.

Ammonia and nitrate were quantified using a SEAL AutoAnalyzer 3HR segmented flow analyzer (Seal Analytical Inc., Mequon, WI). Ammonia and nitrate assays followed standard protocols as per the manufacturer’s instructions (no. G-102-93 for ammonia and no. G-109-94 for nitrate). Further details are described by Johnston et al. (32).

### Data availability.

16S rRNA and *amoA* gene and transcript sequences are available from the NCBI Sequence Read Archive under accession numbers PRJNA591266 and PRJNA605150, respectively.
