# Supplementary material for: Ammonia-Oxidizing Bacteria Maintain Abundance but Lower amoA-Gene Expression during Cold Temperature Nitrification Failure in a Full-Scale Municipal Wastewater Treatment Plant
Source: Microbiol Spectr. 2023 Feb 14;11(2):e02571-22. doi: 10.1128/spectrum.02571-22 (PMC10100873; doi:10.1128/spectrum.02571-22)
Supplement: Supplemental file 1 — Supplemental material. Download spectrum.02571-22-s0001.pdf, PDF file, 1.5 MB [file spectrum.02571-22-s0001.pdf]

Ammonia-oxidizing bacteria maintain abundance but lower *amoA*-gene expression during cold temperature nitrification failure in a full-scale municipal wastewater treatment plant

Supplemental Material

Juliet Johnston[1,2], Zhe Du[1,3], and Sebastian Behrens[1,4*]

[1] University of Minnesota, Department of Civil, Environmental, and Geo-Engineering, 500 Pillsbury Drive S.E, Minneapolis, MN 55455-0116, USA

[2] Lawrence Livermore National Laboratory, Physical and Life Sciences Directorate, 7000 East Ave, Livermore, CA 94550, USA

[3]Center for Environmental Health Risk Assessment and Research, Chinese Research Academy of Environmental Sciences, Beijing 100012, China

[4] University of Minnesota, BioTechnology Institute, 1479 Gortner Avenue, St. Paul, MN 55108-6106, USA

***Corresponding Author:**
Sebastian Behrens
University of Minnesota
BioTechnology Institute
Snyder Hall #338
1479 Gortner Avenue
St. Paul, MN 55108-6106
Phone: (651) 756-9359
e-mail: sbehrens@umn.edu

	Summer '17	Fall '17	Winter '17	Spring '18
**Sample Date**	July 1st, 2017	October 3rd, 2017	December 27th, 2017	March 27th, 2018
**Inf. Water Temp. (°C)**	17.53 ± 0.51	15.93 ± 0.91	13.89 ± 0.90	11.56 ± 0.58
**Air Temp. Range (°C)**	13.3 to 27.8	11.7 to 16.1	-33.9 to -20.6	-18.8 to -8.1
**Influent (m^3/day)**	9 774 ± 318	12 911 ± 1 591	9 956 ± 409	9 410 ± 364
**Effluent (m^3/day)**	8 910 ± 455	12 547 ± 1 818	9 138 ± 591	8 638 ± 409
**Inf. BOD5 (mg/L)**	175 ± 52	167 ± 64	139 ± 48	164 ± 29
**Eff. BOD5 (mg/L)**	2.00 ± 0.00	1.23 ± 0.60	1.33 ± 0.49	1.75 ± 0.27
**Inf. TSS (mg/L)**	338 ± 138	378 ± 261	169 ± 52	263 ± 47
**Eff. TSS (mg/L)**	2.83 ± 1.34	2.85 ± 2.41	1.92 ± 0.67	2.92 ± 1.38
**Inf. Phos. (mg/L)**	4.87 ± 0.44	3.58 ± 0.67	5.08 ± 0.30	5.51 ± 0.47
**Eff. Phos (mg/L)**	0.20 ± 0.06	0.33 ± 0.23	0.15 ± 0.06	0.18 ± 0.02
**Eff. NH3 (mg/L)***	0.10	0.10	4.12	9.43
**MLSS (mg/L)**	1 378 ± 166	1 335 ± 152	1 165 ± 219	1 092 ± 167

*Values only recorded once per month. Date closest to sampling trip was used.

Table S1 features seasonal operational conditions and performance metrics for the sample trips taken throughout 2017-2018. The data presented is an average of a 30-day window around the sample period. The influent water and air temperatures, and flowrates, had periods of statistically significant differences compared to other seasons. Other than effluent ammonia, typical effluent operational performance metrics for biological oxygen demand, phosphorous, and total suspended solids did not statistically vary throughout the seasons. This data was also reported in (Johnston and Behrens, 2020).

Mixed	Summer	Fall	Winter	Spring
Summer	x			
Fall	1.23026E-06	x		
Winter	0.969734789	7.27873E-07	x	
Spring	0.000179126	9.3652E-14	4.7604E-05	x

React 1	Summer	Fall	Winter	Spring
Summer	x			
Fall	0.000685039	x		
Winter	0.169468207	6.81654E-05	x	
Spring	0.000413283	2.67945E-08	0.03756967	x

Settled Sludge	Summer	Fall	Winter	Spring
Summer	x			
Fall	0.00033102	x		
Winter	0.204093551	0.002259162	x	
Spring	5.65888E-07	3.45204E-11	0.026474	x

React 2	Summer	Fall	Winter	Spring
Summer	x			
Fall	0.000529889	x		
Winter	0.736656525	0.002615471	x	
Spring	0.531708339	3.2719E-07	0.92071556	x

Decanted Water	Summer	Fall	Winter	Spring
Summer	x			
Fall	0.082595675	x		
Winter	0.27916845	0.019666441	x	
Spring	0.005832032	1.50478E-05	0.266732	x

Table S2 are p-values of *amoA* transcript abundances via RT-qPCR compared throughout different reactor cycles and seasons. Each comparison is from the triplicate sequencing batch reactors over during a 30-minute time frame (ex: 140-minutes, 150-minutes (R1), and 160-minutes) for better statistical accuracy.

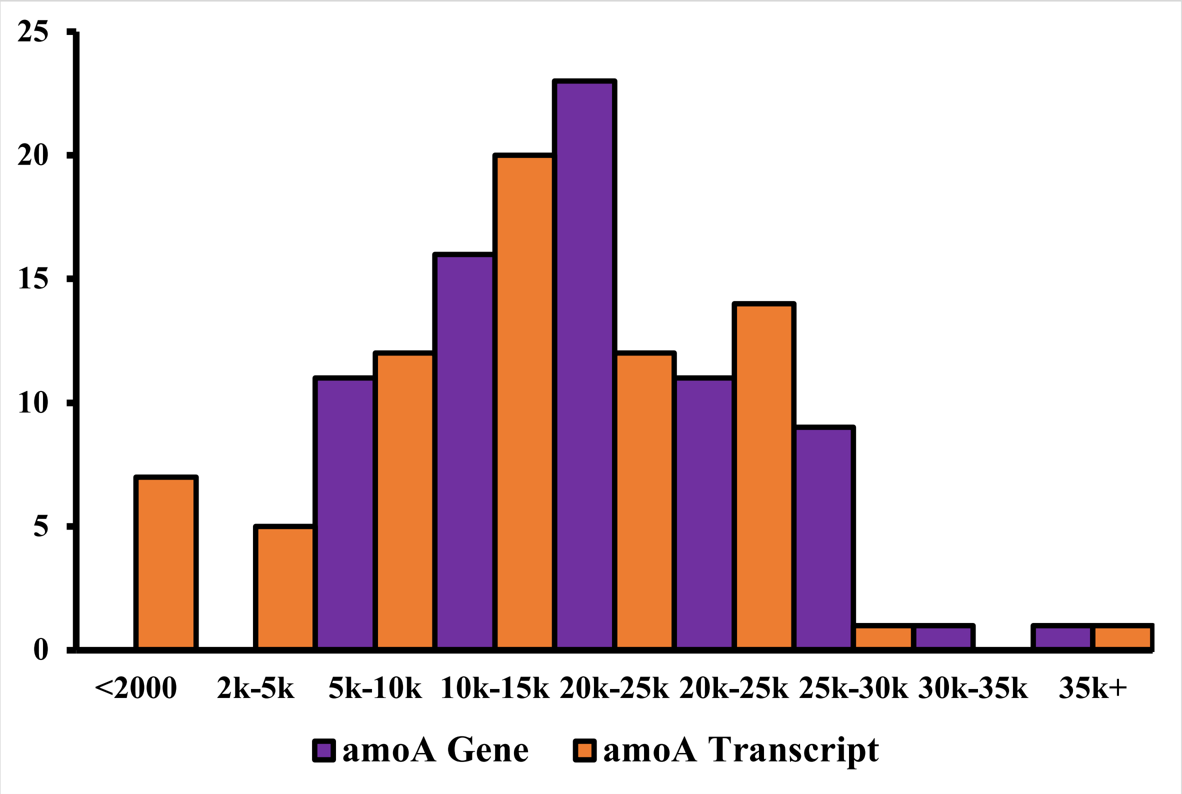

Figure S1 The histogram shows the average distribution of processed ASVs for the 72 *amoA* gene amplicon sequences and the 72 *amoA* transcript amplicon sequences. The *amoA* genes are shown in the purple while the *amoA* transcripts are in orange. The *amoA* gene samples had an average of 16,978±6763 merged pairs per sample while the *amoA* transcript samples averaged 13,106±7431 merged pairs per sample.

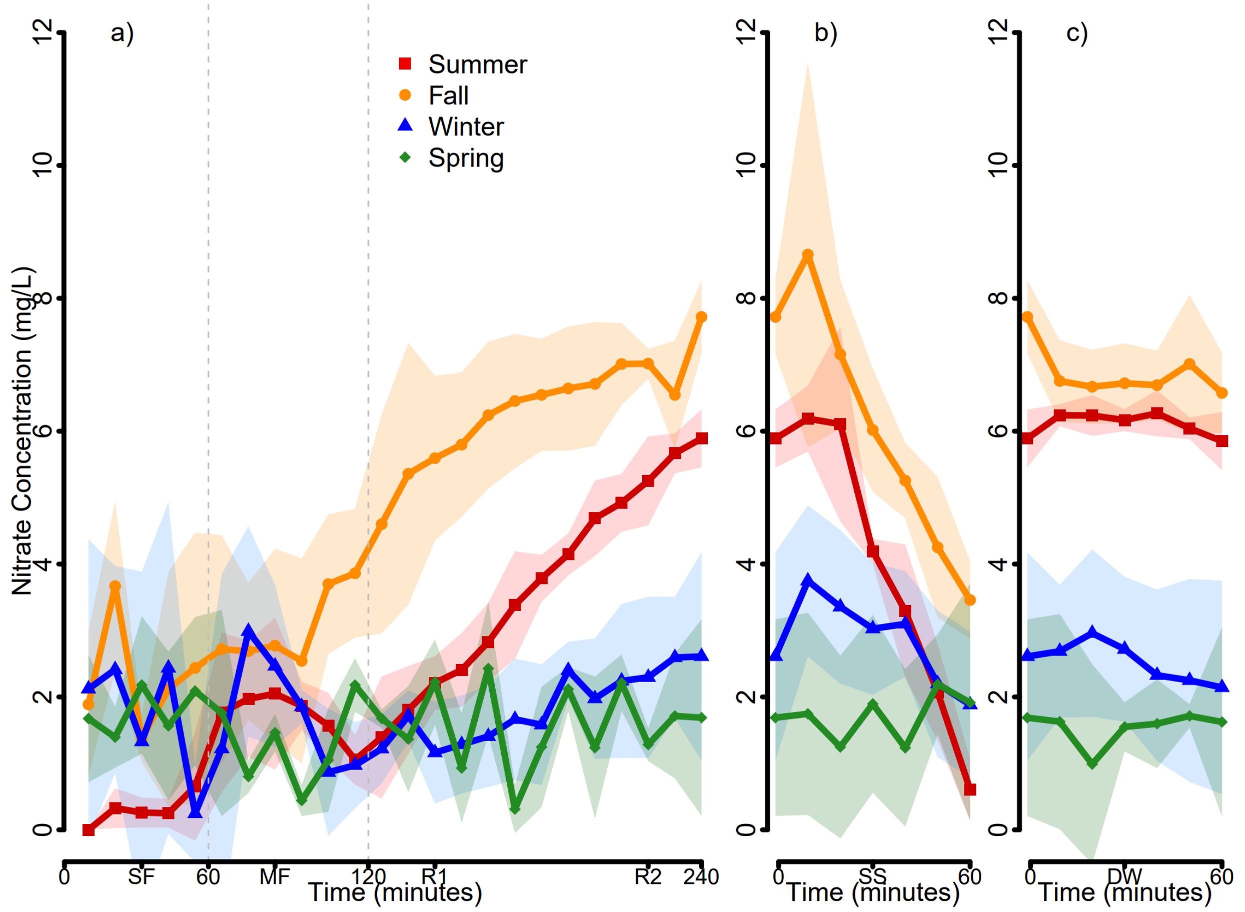

Figure S2 Average nitrate concentration for summer 2017 (red squares), fall 2017 (orange circles), winter 2017 (blue triangles), and spring 2018 (green diamonds). Standard deviations for each season are shown as shaded areas of the respective season color. A. Average nitrate concentrations during the first 240 min of a complete reaction cycle. B. Average nitrate concentrations in the settled sludge during a 60 min settling phase (SS). C. Average nitrate concentrations in the decanted water (effluent supernatant) (DW) above the settled sludge during a 60 min settling phase. Data of panel B and C were recorded simultaneously from samples collected from the bottom (settled sludge) and top (decant effluent water) of each sequencing batch reaction during the 60 min settling phase.

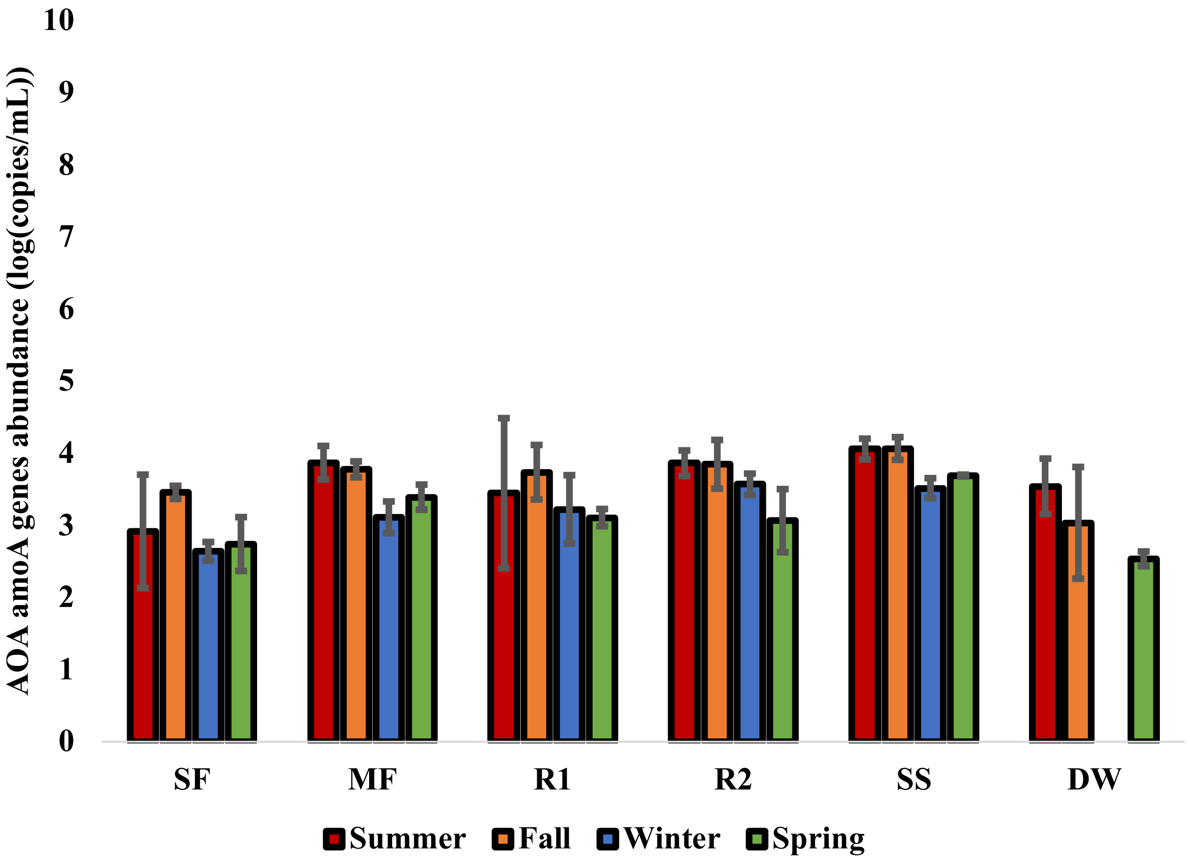

Figure S3 The average abundance of *amoA* genes for ammonia oxidizing archaea were quantified with the standard deviation of the triplicate sequencing batch reactors show. Each color represents a different season throughout the six-time points during the reactor cycle (SF= Static Fill, MF= Mixed Fill, R1 = React 1, R2 = React 2, SS = Settled Sludge, DW = Decanted Water) with summer in red, fall in orange, winter in blue, and spring in green. Throughout the reaction cycle, ammonia oxidizing archaea were about 2.5 orders of magnitude lower than the ammonia oxidizing bacteria. This low abundance of ammonia oxidizing archaea based on *amoA genes* in conjunction with most *amoA* transcript data falling below detection limits was determined to be insignificant overall to wastewater operations and not further analyzed. All *amoA* genes for ammonia oxidizing archaea in the decanted water (DW) for winter were below detection limits.

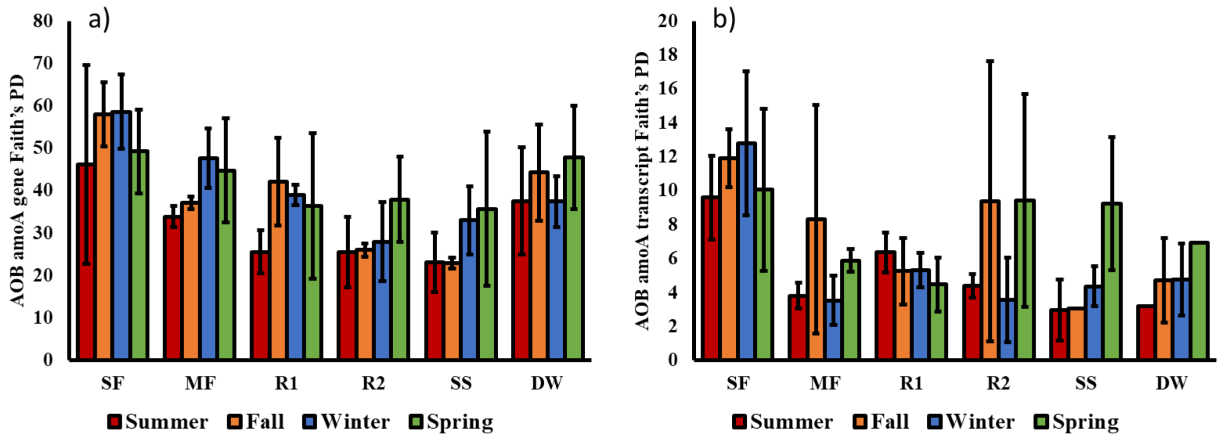

Figure S4 The Faith Phylogenetic Diversity Index was calculated for the *amoA* gene (Figure S3a) and the *amoA* transcript (Figure S3b) are rarified subsamples of 5000 reads and 2000 reads respectively. Each season is depicted throughout various points in the reactor sequence (SF= Static Fill, MF= Mixed Fill, R1 = React 1, R2 = React 2, SS = Settled Sludge, DW = Decanted Water) with summer in red, fall in orange, winter in blue, and spring in green. The triplicate sequencing batch reactors were averaged together after taking an individual reactors average over 1000-bootstrapped iterations. The standard deviations represent only the average between the three individual reactors, not the bootstrapped values. Even when the *amoA* gene was rarified to 2000 reads to be more comparable to *amoA* transcripts, the overall phylogenetic diversity of *amoA* genes is consistently higher than *amoA* transcripts. Additionally, when averaging the seasons together, the static fill (SF) is always statistically higher in diversity than the other reactor time points.

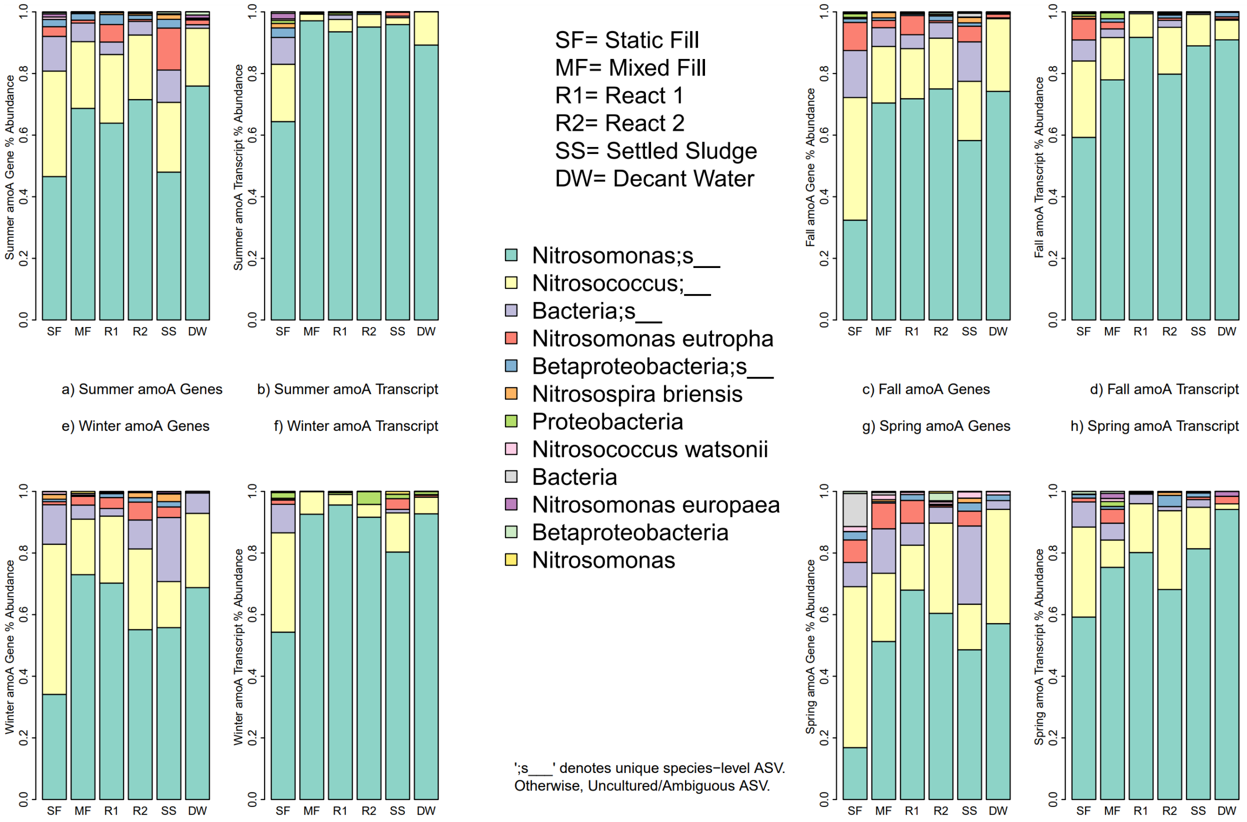

Figure S5 The average relative abundance of the triplicate sequencing batch reactors ASV clusters per season are represented above with summer in the upper left, fall in the upper right, winter in the lower left, and spring in the lower right. Each season's quadrant has both the relative abundance of ASV clusters for *amoA* genes on the left, and *amoA* transcripts on the right. Additionally, the reactor sequence is noted along the x-axis of each histogram (SF= Static Fill, MF= Mixed Fill, R1 = React 1, R2 = React 2, SS = Settled Sludge, DW = Decanted Water). *Nitrosomonas* dominates the activated sludge system overall, while *Nitrosococcus* was the most abundant cluster in the influent wastewater during static fill (SF).

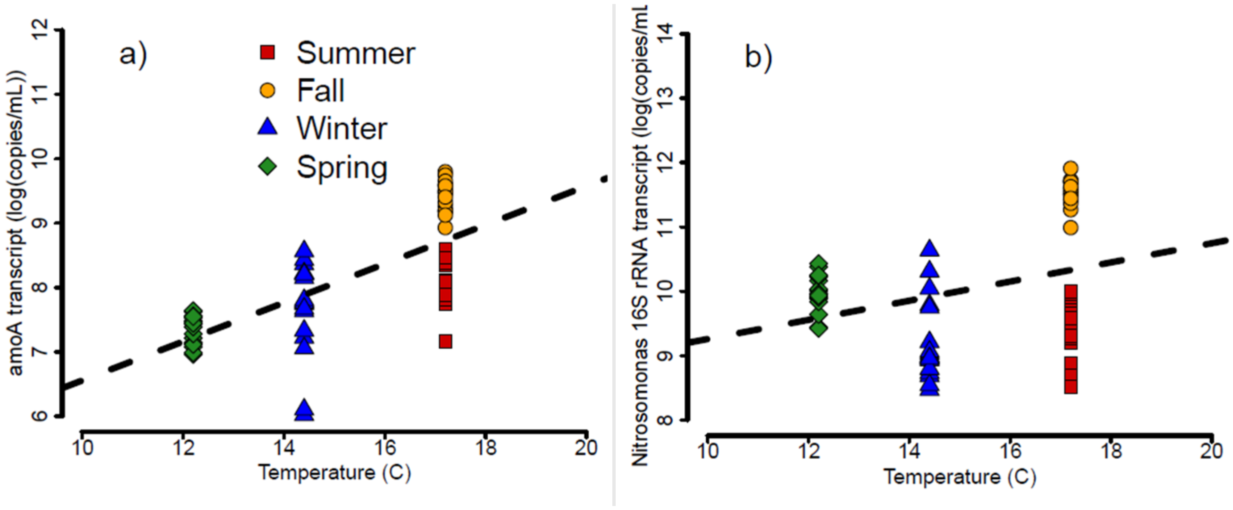

Figure S6. Linear regression of influent wastewater temperature throughout the seasons with Log-normalized a) amoA transcript copy numbers and b) *Nitrosomonas* 16S rRNA transcript copy numbers. All data points were taken during the aerobic react cycle phase to determine the correlation between temperature and transcript abundances. Summer is represented in red squares, fall in orange circles, winter in blue triangles and spring in green diamonds. The equations for each respective correlative regression is a) *amoA* transcript abundance = Y = 0.302 * (X°C) + 3.540 with an R^2=0.50 and b) Nitrosomonas 16S rRNA transcripts = Y = 0.149 * (X°C) + 7.773 with an R^2=0.098.

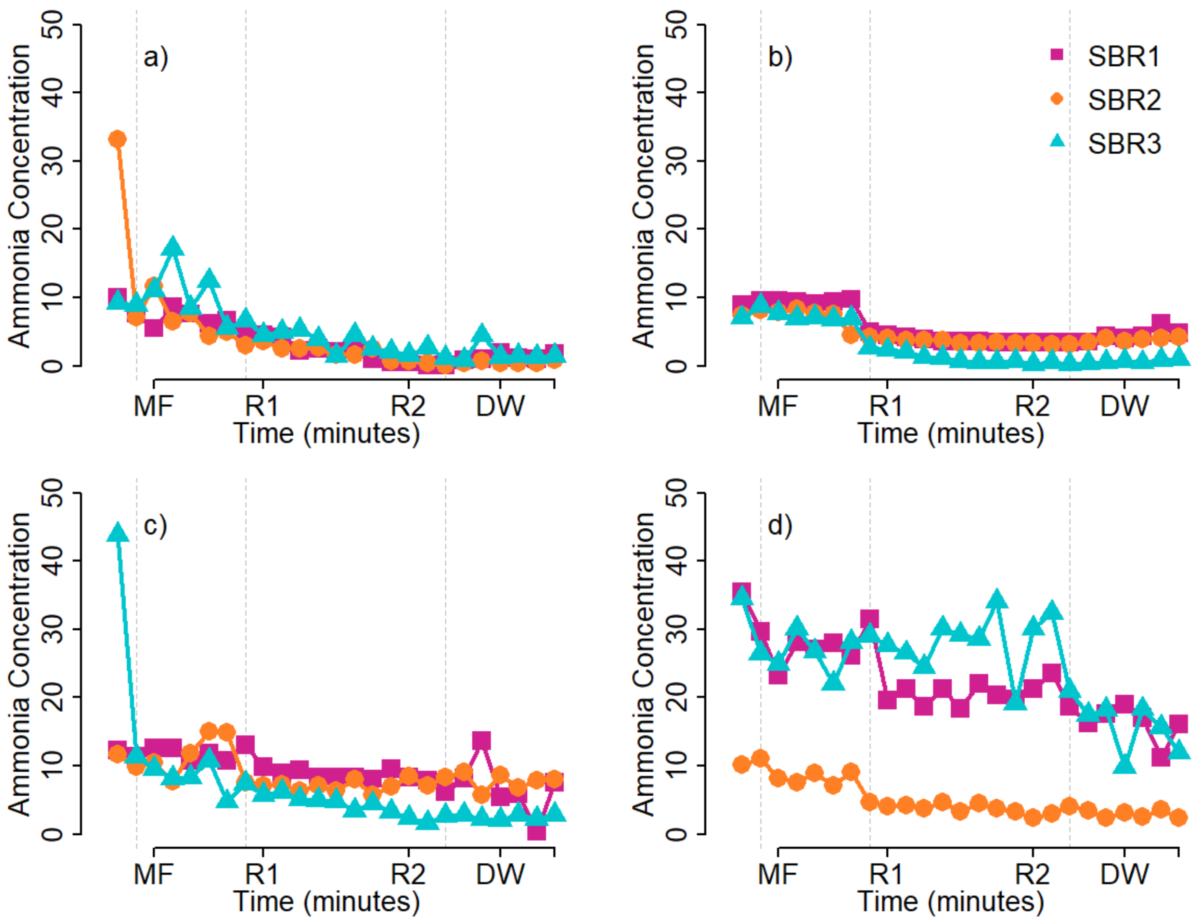

Figure S7 The ammonia concentration is shown throughout the triplicate sequencing batch reactors with each plot focusing on a different season. The plots are continuous from the end of static fill, through the hour-long mixed fill (MF), into the two-hour aerobic react cycle (R1 & R2), and through settling of the water about to be decanted from the reactor (DW). The reactors in summer are shown in the top left (a), fall is the top right (b), winter is the bottom left (c), and spring in the bottom right (d). Each reactor is color coded with sequencing batch reactor 1 (SBR1) in violet, reactor 2 in orange (SBR2), and reactor 3 (SBR3) in turquoise.
